# Phenolic compounds of sugar beet (*Beta vulgaris* L.): Separation method, chemical characterization, and biological properties

**DOI:** 10.1002/fsn3.3017

**Published:** 2022-09-13

**Authors:** Edris Arjeh, Seyedeh Mahsa Khodaei, Mohsen Barzegar, Sajad Pirsa, Iraj Karimi Sani, Shiva Rahati, Farzad Mohammadi

**Affiliations:** ^1^ Department of Food Science and Technology, Faculty of Agriculture Urmia University Urmia Iran; ^2^ Faculty of Nutrition and Food Sciences Isfahan University of Medical Sciences Isfahan Iran; ^3^ Department of Food Science and Technology, Faculty of Agriculture Tarbiat Modares University Tehran Iran; ^4^ Department of Nutrition, Faculty of Medicine Mashhad University of Medical Sciences Mashhad Iran; ^5^ Afagh Higher Education Institute Urmia Iran

**Keywords:** antioxidant, *Beta vulgaris* L., chelating ability, phenolic compound, sugar beet

## Abstract

Sugar beet (*Beta vulgaris* L.) is a good source of bioactive compounds. However, information on the biological properties of sugar beet root is limited and its beneficial effects have not been completely understood. In this work, 10 phenolic compounds have been separated and identified in various parts of sugar beet for the first time, including the most abundant epicatechin (31.16 ± 1.89 mg/100 g), gallic acid (30.57 ± 2.69 mg/100 g), and quercetin‐3‐O‐rutinoside (30.14 ± 3.63 mg/100 g). The biological activity tests indicated that sugar beet peel potently scavenged the nitric oxide and DPPH (2,2‐diphenyl‐1‐picrylhydrazyl) free radicals with IC_50_ values of 88.17 ± 05.14 and 28.77 ± 0.62 μg/ml, respectively. In addition, sugar beet peel exhibited the highest reducing power, IC_50_ values of 11.98 ± 1.20 μg/ml, and the highest ion‐chelating activity, IC_50_ values of 48.52% and 55.21% for cupric and ferrous ions at 250 μg/ml, respectively. Compared to synthetic antioxidants, sugar beet showed promising biological activities, which could be considered further in future studies.

## Graphical abstract

Sugar beet is a rich source of bioactive compounds. Ten polyphenolic compounds were identified for the first time in sugar beet parts. Epicatechin, gallic acid, and rutin were the major phenolic components. Peel extract of sugar beet exhibited strong antioxidant and chelating activity.

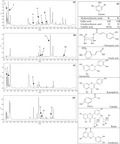

## 1 INTRODUCTION

Sugar beet (*Beta vulgaris* L.) is a biennial plant that originated from the Mediterranean region and belongs to the *Chenopodiacea* family. The root of sugar beet is an abundant source of bioactive compounds, minerals, and vitamins (Filipčev et al., 2010; Koprivica et al., 2014; Mikołajczyk‐Bator et al., 2016), which provides health‐promoting effects such as the inhibition of tumor cell growth (Valli et al., 2012), protection from the oxidative damage (Filipčev et al., 2010; Mohdaly et al., 2010), and prevention of the age‐related diseases (Drobny et al., 2020). In Iranian traditional medicine, sugar beet has been considered as a medicinal root and is used to treat the psychiatric problems and strengthen the human immune system (El‐Gendy et al., 2022). However, sugar beet is mainly processed for sugar production which leads to the loss of health‐promoting compounds.

Phenolic compounds are the most widely present secondary metabolites in the plant tissues, ranging from a diversity of structures, from simple molecules, such as phenolic acids, to polyphenols such as flavonoids, which comprise several groups (Nollet & Gutierrez‐Uribe, 2018). In recent years, phenolic compounds have attracted much attention in the food and pharmaceutical industry for several reasons (Dias et al., 2021; Zeb, 2020). Phenolic compounds can act as reducing agents against the reactive oxygen/nitrogen species that are proposed to cause oxidative damage in the body’s tissue (Huang & Tocmo, 2018). Therefore, they exhibit a wide/broad range of biological properties such as antimicrobial, antiviral, anti‐inflammatory, anti‐allergenic, antithrombotic, cardioprotective, and vasodilatory effects (Chen et al., 2017; Singh et al., 2017).

Despite the importance of phenolic compounds in the nutrition issues and pharmaceutical industry, studies on the phenolic profile of sugar beet root are limited, and its phenolic composition has not yet been completely identified. The total phenolic content (TPC) of the sugar beet peel (*Beta vulgaris altissima*) was reported as 4.2 mg/g dw by Kähkönen et al. (1999). They also indicated that the sugar beet peel had a remarkable antioxidant activity (88% inhibition) compared to the other vegetable sources. Also, the high antifungal activity of sugar beet extract is attributed to the presence of a high amount of ferulic acid and hydroxycinnamic acid‐type phenolics (Nagahashi et al., 1996). Therefore, due to lack of adequate knowledge about biological activity and phenolic compositions of different parts of sugar beet, this work was carried out to separate and identify the phenolic compounds of sugar beet flesh and peel. In addition, for estimation of the biological activity, the antioxidant and chelating capacity of flesh and peel of sugar beet was determined.

## 2 MATERIALS AND METHODS

### 2.1 Chemicals

Folin–Ciocalteu reagent and N‐(1‐naphthyl)ethylenediamine were purchased from Merck Chemical Co. DPPH (2,2‐diphenyl‐1‐picrylhydrazyl), trolox (6‐hydroxy‐2,5,7,8‐tetramethylchromane‐2‐carboxylic acid), EDTA (ethylenediaminetetraacetic acid), BHT (butylated hydroxytoluene), sodium nitroprusside (Na_2_[Fe[CN]_5_NO]), epicatechin, gallic acid, quercetin‐3‐O‐rutinoside, kaempferol, eriodictyol, 4‐hydroxybenzoic acid, chlorogenic acid, vanillin, vanillic acid, and ferulic acid were purchased from Sigma‐Aldrich. TPTZ (2,4,6‐Tris[2‐pyridyl]‐s‐triazine) was purchased from Acros Organics Company. Gelatin (type A; 100 bloom) was purchased from Erbigel Company. Other chemicals and solvents were of analytical grade.

### 2.2 Preparation of raw materials

Sugar beet roots (*Beta vulgaris* L. cv. ISABELLA produced by KWS Company) were harvested in the 2017 vegetative seasons from a commercial farm, Piranshahr, West Azerbaijan province, Iran. The roots were randomly sampled and washed with rinse water. The roots were then hand peeled and sliced with sharp knife. The peel and flesh were separately lyophilized using a freeze dryer at 1 mbar and −50°C for 24 h (Alpha 1‐2 LDplus). The freeze‐dried samples were ground using a laboratory mill and stored at −20°C until extraction.

### 2.3 Extraction methods

Ground samples were first defatted by stirring for 30 min in diethyl ether at room temperature with a powder/solvent ratio of 1:10 (w/v). Five grams of residue was mixed with 100 ml of solvent (methanol/ethanol) at room temperature and subjected to bath sonication amplitude (James Products, Model 6D) for 20 min. The extraction continued under stirring for 4 h (100 ml × 3 times). The combined extract was then centrifuged at 2000 *g* for 10 min and the supernatant was filtered through Whatman No. 1 filter paper and concentrated to dryness using a rotary evaporator (Heidolph, model Laborota 4001). Then, the dried extract was dissolved in 1 ml of solvent and filtered through a syringe filter (0.22 μm, MS nylon syringe filter) into vials prior to analyses. The vials were wrapped in an aluminum foil to protect from light.

### 2.4 Total phenolic content assay

The TPC was determined according to the procedure described by Seifzadeh et al. (2019) using the Folin–Ciocalteu reagent and the results were expressed as milligrams (mg) of gallic acid equivalent (GAE) per gram of ground powder (mg/g db). Briefly, 20 ml of the samples was added to 1.4 ml H_2_O and 100 μl of the Folin–Ciocalteu reagent. After shaking for 5 min, 0.3 ml of a solution (75 g/L) of sodium carbonate (Na_2_CO_3_) was added and the mixture was incubated at 40°C for 30 min, with continuous stirring. The absorbance (resulting blue complex) was immediately read at 765 nm using a UV‐2100 spectrophotometer (SCINCO). Standard solutions of gallic acid (100–600 ppm) were treated similarly to prepare the calibration curve (*y* = 0.0008 × *x* + 0.138, *R* ^2^ = .98).

### 2.5 Total flavonoids content assay

The total flavonoids content was determined according to the procedure described by Heimler et al. (2005) with slight modifications. Briefly, 250 μl of the extract was mixed with 75 μl of a 5% solution of sodium nitrite (NaNO_2_). After 5 min, 150 μl of 10% solution of aluminum chloride (AlCl_3_) was added and allowed to react at room temperature for another 5 min. Subsequently, 500 μl of sodium hydroxide (NaOH, 1 N) was added and brought to 3 ml with distilled water. The absorbance was immediately measured at 510 nm using a UV‐2100 spectrophotometer (SCINCO). Standard solutions of gallic acid (20–400 ppm) were treated similarly to prepare the calibration curve (*y* = 0.0015 × *x* + 0.121, *R* ^2^ = .98).

### 2.6 Determination of the total tannins content

The total tannins content (TTC) was determined by the gelatin method previously described by Ramdane et al. (2017) and the results were expressed as milligrams (mg) of GAE per gram of ground powder (mg/g db). Briefly, 200 mg of the gelatin was mixed with 2 ml of water and 2 ml of extract and shaken vigorously. The mixture was then incubated at 4°C for 15 min. Finally, the mixture was centrifuged and filtered through Whatman No. 1 filter paper. Nontannin phenolics were determined using the Folin–Ciocalteu reagent and the TTC of the extract was calculated by subtracting nontannin phenolics from the TPC.

### 2.7 Separation and quantification of polyphenolic compounds by RP‐HPLC‐UV

Phenolic compounds of extracts were determined using high performance liquid chromatography (HPLC) (Waters) in an Empower software and UV–visible (UV–vis) detector (Waters model 2487) at 285 and 340 nm according to the procedure previously described by Barreca et al. (2016). An ODS‐UG‐5 reversed‐phase column (Develosil 120‐5‐C18 AQ, 250 × 4.6 mm, 5 μm, Nomura Chemical Co., Ltd.) was applied for the separation of phenolics. The injection volume was 20 μl using an injector with a 20 μl loop. The elution was carried out at room temperature using acidified water (solvent A) containing 3% acetic acid (V/V) and methanol (solvent B) at the flow rate of 0.1 ml/min as the mobile phase. The solvent gradient system was as follows: isocratic at 0% B for 3 min, from 0% to 3% B in 6 min; from 3% to 12% B in 15 min; from 12% to 20% B in 6 min; isocratic at 20% B for 3 min; from 20% to 30% B in 10 min; from 30% to 50% B in 20 min; from 50% to 60% B in 10 min; isocratic at 60% B for 5 min; from 60% to 0% B in 10 min and equilibrated 14 min for a total run time of 100 min. Calculation of the concentrations was based on the external standard method and phenolic compounds were identified by their retention times (Guclu et al., 2021). Results were expressed as grams (g)/100 g of the dried powder.

### 2.8 Determination of antioxidative activity of sugar beet

#### 2.8.1 DPPH free radical scavenging activity

The free radical scavenging activity of the extracts was determined using the stable 2,2‐diphenyl‐1‐picrylhydrazyl radical (DPPH٠) according to the procedure described by Seifzadeh et al. (2018). Briefly, 2.7 ml of 0.1 mM methanolic solution of DPPH radicals was added to the different concentrations of the samples (300 μl). The mixtures were incubated at room temperature for 30 min. The absorbance was then measured at 517 nm using a UV‐2100 spectrophotometer. The ability of scavenging was calculated using the following formula:

$$\text{Scavenging activity}\;\left(\%\text{inhibition}\right)=\left[\left(A_{C}-A_{S}\right)/\left(A_{C}\right)\right]\times 100$$

Where *A*_C_ and *A*_S_ are the absorbance values of the control and extract samples at 517 nm, respectively. The IC_50_ value is the effective concentration (μg/ml) at which the DPPH radicals were scavenged by 50% and were calculated by interpolation from linear regression analysis.

#### 2.8.2 Ferric reducing antioxidant power

The reducing power of the extracts was determined using the 2,4,6‐Tris[2‐pyridyl]‐S‐triazine (TPTZ) according to the procedure described by Benzie and Devaki (2018) with some modifications. First, the fresh ferric reducing antioxidant power (FRAP) reagent was prepared by mixing TPTZ (10 mM in HCl 40 mM), acetate buffer (300 mM, pH = 3.6), and iron (III) chloride hexahydrate (FeCl_3_.6H_2_O, 20 mM) at a ratio of 1:10:1 v/v/v. Then, 200 μl of different concentrations of samples was mixed with 3.8 ml of the FRAP reagent and allowed to react in dark at 37°C for 30 min. The increase in absorbance was recorded at 593 nm on a UV‐2100 spectrophotometer. The antioxidant power was calculated using the following formula:

$$\text{Antioxidant activity}\;\left(\mathrm{AoA},\%\right)=\left[\left(A_{C}-A_{S}\right)/\left(A_{C}\right)\right]\times 100$$

Where *A*_C_ and *A*_S_ are the absorbance values of the control and samples at 593 nm, respectively.

#### 2.8.3 Nitric oxide radical scavenging assay

The nitric oxide radical scavenging (NOs) activity of the extracts was determined using the Griess reagent (1% sulfanilamide in 5% phosphoric acid (H_3_PO_4_) and 0.1% N‐(1‐naphthyl)ethylenediamine in distilled water in equal volumes) according to the procedure described by Huang and Tocmo (2018). Briefly, sodium nitroprusside (Na_2_[Fe(CN)_5_NO]) in phosphate‐buffered saline (10 mM, pH = 7.3) was added to different concentrations of samples in a ratio of 1:1 and incubated for 30 min at room temperature. Next, 300 μl of incubated solution was mixed with 300 μl of Griess reagent and the absorbance was measured at 540 nm after 10 min incubation. The NOs activity was calculated using the following formula:

$$\text{Scavenging activity}\;\left(\%\text{inhibition}\right)=\left[\left(A_{C}-A_{S}\right)/\left(A_{C}\right)\right]\times 100$$

Where *A*_C_ and *A*_S_ are the absorbance values of the control and extract samples at 540 nm, respectively.

### 2.9 Chelating ability of ions

#### 2.9.1 Ferrous ions chelation assay

The chelating ability of the samples for ferrous ions was determined according to the procedure described by Abolhasani et al. (2018). First, 50 μl of 0.6 mM solution of FeCl_2_ was added to the different concentrations of the samples (700 μl). After that, 50 μl of 5 mM solution of ferrozine was added to the mixture and shaken vigorously. The mixture was incubated for 10 min at room temperature. The absorbance was then measured at 562 nm using a UV‐2100 spectrophotometer. The chelating ability of ferrous ions was calculated using the following formula:

$$\text{Ferrous ions chelating activity}\;\left(\%\text{inhibition}\right)=\left[\left(A_{C}-A_{S}\right)/\left(Ac\right)\right]\times 100$$

Where *A*_C_ and *A*_S_ are the absorbance values of the control and extract samples at 562 nm, respectively. The IC_50_ value is the effective concentration (μg/ml) at which the ferrozine–Fe^2+^ complex was inhibited by 50% and was calculated by interpolation from linear regression analysis.

#### 2.9.2 Cupric ions chelation assay

The chelating ability of the samples for cupric ions was determined according to the procedure described by Wong et al. (2006) with slight modifications. First, a mixture consisting of hexamine (30 mM), potassium chloride (KCl, 30 mM), and copper (II) sulfate (CuSO_4_, 9 mM) was made. One ml of the prepared mixture was mixed with 1 ml of the various concentrations of methanolic extracts. Thereafter, 100 μl of tetramethylmurexide ammonium salt (2 mM) was added and shaken vigorously. The absorbance was then measured at 460 and 530 nm using a UV‐2100 spectrophotometer and the ratio between them (*A*_460_/*A*_530_) was determined. This ratio was then converted to corresponding free Cu (II) concentrations by comparing with a standard curve of free Cu (II) concentration. By reducing the amount of free copper (II) from the total amount, the concentration of chelated cupric ions was obtained. The chelating ability of cupric ions was calculated using the following formula:

$$\text{Cupric ions chelating activity}\;\left(\%\text{chelating}\right)=\left[\mathrm{CC}/\mathrm{TC}\right]\times 100$$

Where CC and TC are the concentrations of the chelated and total cupric ions, respectively.

## 3 RESULTS AND DISCUSSION

### 3.1 Determination of total phenolic, flavonoids, and tannins contents

Results showed that the root peel extracts had higher values for all the conducted experiments compared to the flesh. These results were in accordance with the study of Kujala et al. (2000), showing that phenolic compounds are distributed mostly to outer parts of the root. The amount of phenolic compounds in the peel (19.7 ± 1.1 g/mg db) was over twice that of the flesh (8.3 ± 0.0 g/mg db). Moreover, solvents also had a significant effect on the quantity of phenolic compounds, and methanol was more effective than ethanol in the extraction of phenolic compounds. Barreca et al. (2016) also reported similar results. It has been illustrated that higher polarity solvents often perform better in polyphenol extraction because polyphenols have higher solubility in such solvents (Alara et al., 2021). TPCs of the methanolic and ethanolic extracts of flesh were 8.3 ± 0.0 mg/g and 5.6 ± 0.5 mg/g db for methanol and ethanol, respectively. Therefore, methanol was selected for subsequent experiments. Table 1 also shows total flavonoids and tannins contents of the root peel and flesh. Flavonoids and tannins are two large classes of phenolic compounds that exhibit a wide spectrum of biological properties (Ng et al., 2020; Sadeghnezhad et al., 2020; Santos et al., 2021). They were found in both parts, although the methanolic extract of peel had a higher value than flesh extracts.

**TABLE 1:** Total phenolic, flavonoids, and tannins contents (milligrams of gallic acid equivalent per gram of ground powder (mg GAE/g db)) of methanolic and ethanolic extracts of different parts of sugar beet

	Flesh		Peel	
	Methanolic extract	Ethanolic extract	Methanolic extract	Ethanolic extract
TPC	8.3 ± 0.0	5.6 ± 0.5	19.7 ± 1.1	9.8 ± 0.2
TFC	3.2 ± 0.1	2.1 ± 0.0	5.8 ± 0.4	3.9 ± 0.3
TTC	3.7 ± 0.5	2.2 ± 0.2	4.3 ± 0.1	3.5 ± 0.4

Abbreviations: GAE, gallic acid equivalent; TFC, total flavonoids content; TPC, total phenolic content; TTC, total tannins content.

### 3.2 Separation and quantification of phenolic compounds of sugar beet

**FIGURE 1:**
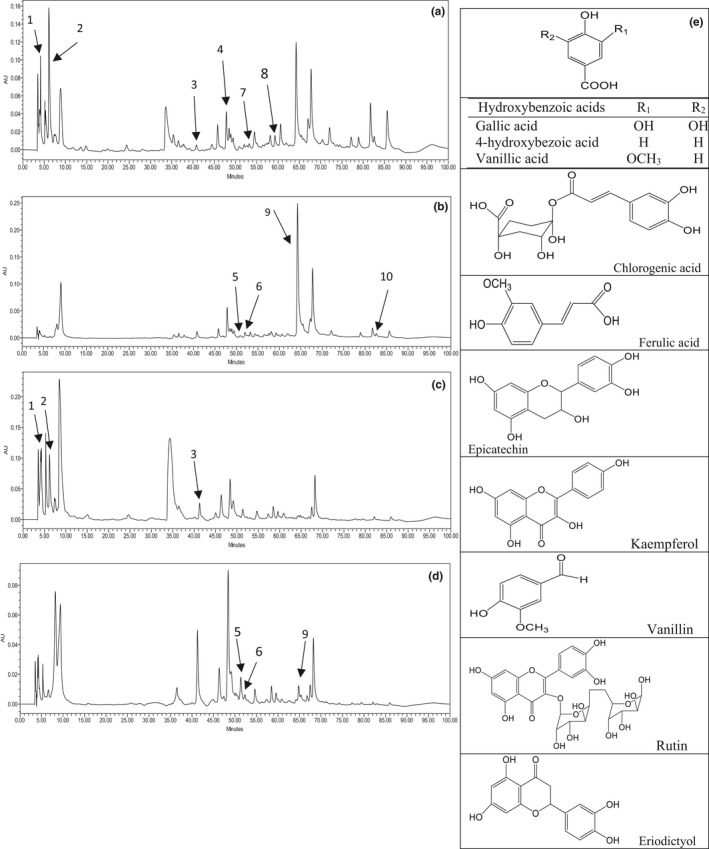
Phenolic compound chromatograms of the methanol extract of (a) beet peel at 285 nm, (b) beet peel at 340 nm, (c) beet flesh at 285 nm, and (d) beet flesh at 340 nm. Peaks are marked with the same numbers presented in Table 2. (e) Chemical structures of the detected phenolics.

Phenolic compounds of peel and flesh of sugar beet root, detected at 280 and 340 nm, are reported in Figure 1. In this study, 10 phenolic compounds were identified: gallic acid, epicatechin, 4‐hydroxybenzoic acid, vanillic acid, vanillin, eriodictyol, chlorogenic acid, ferulic acid, quercetin‐3‐O‐rutinoside, and kaempferol. Based on our knowledge, this is the first time that phenolic compounds have been separated and identified in sugar beet.

Table 2 presents the concentration of the identified phenolics of the sugar beet. The phenolics existing in the methanolic extracts are quantified by well‐defined peaks with maximum absorbance at 340 nm for flavonoids and at 285 nm for phenolic acids. The remarkable differences between phenolic compounds of peel and flesh extracts are due to the absence or presence of chlorogenic acid and kaempferol, and to the relative concentration of them. Vanillin, vanillic acid, hydroxybenzoic acid, ferulic acid, and kaempferol are the phenolic compounds previously identified in sugar beet by‐products (molasses and pulp) (Valli et al., 2012), but other phenolic compounds (gallic acid, epicatechin, eriodictyol, chlorogenic acid, and quercetin‐3‐O‐rutinoside) were unambiguously identified for the first time among sugar beet and its by‐products (pulp and molasses) extracts. As can be seen, epicatechin, gallic acid, and quercetin‐3‐O‐rutinoside are the dominant phenolics that are detected in sugar beet and presented average amounts of 31.16 ± 1.89, 30.57 ± 2.69, and 30.14 ± 3.63 mg/100 g in peel extract, respectively. Gallic acid (3,4,5‐trihydroxybenzoic acid) is a low molecular weight phenolic compound that is widely found in plants and can be either free or as part of hydrolyzable tannins. Nayeem et al. (2016) stated that gallic acid and its derivatives can exhibit their potential to prevent various diseases, such as ischemic heart diseases, diabetes, cancer, and other ailments. Rutin (quercetin‐3‐O‐rutinoside) is one of the most prominent antioxidative flavonoids that scavenge free radical by its phenolic hydroxyl groups at the B‐ring and the 3‐position (Murakami et al., 2008). Numerous studies have illustrated that quercetin derivatives have a high ability to scavenge the reactive species such as the hydroxyl and peroxynitrite (Amiri et al., 2021; Boots et al., 2008). Epicatechin is another dominant phenolic compound which is widely found in tea and belongs to the group of flavan‐3‐ols. Researchers have attributed a wide range of biological activities to epicatechin, such as antioxidant, antibacterial, hypoglycemic, insulin resistance, as well as preventing anti‐inflammatory, enhancing immunity, cardiovascular disease, and so on (Tong et al., 2018). The HPLC analyses also showed that the peel extract had a higher amount of phenolics than the flesh extract (Table 2), which confirms our total phenolic assay results.

**TABLE 2:** Phenolic compounds detected in methanolic extract of sugar beet. Data are presented as mg/100 g dried powder

Compounds	Peak no.	RT (min)	Phenolic content	
			Peel	Flesh
Hydroxybenzoic acids				
Gallic acid	1	4.3	30.57 ± 2.69	22.95 ± 1.35
4‐hydroxybenzoic acid	3	41.2	4.36 ± 1.02	6.02 ± 0.70
Vanillic acid	4	48.3	1.87 ± 0.49	1.52 ± 0.18
Hydroxycinnamic acids				
Chlorogenic acid	7	53.2	2.24 ± 0.21	nd
Ferulic acid	8	59.9	3.20 ± 0.00	1.06 ± 0.03
Hydroxybenzoic acid				
Vanillin	5	51.1	0.65 ± 0.13	0.99 ± 0.20
Flavanol				
Epicatechin	2	6.3	31.16 ± 1.89	10.57 ± 1.27
Flavanone				
Eriodictyol	6	52.3	0.52 ± 0.00	0.26 ± 0.03
Flavonols				
Quercetin‐3‐O‐rutinoside	9	64.6	30.14 ± 3.63	1.46 ± 0.06
Kaempferol	10	82.9	12.15 ± 1.62	nd

Abbreviations: nd, not detected; RT, retention time.

### 3.3 Determination of antioxidant activity

In the present study, the antioxidant activities of sugar beet extracts and related phenolics were analyzed by three different in vitro methods to obtain a clear perspective from the antioxidant power of sugar beet. As can be seen in Table 3, both flesh and peel extracts exhibit high reducing power and radical scavenging activity toward Fe^3+^ and DPPH٠, although the antioxidant activity of peel was clearly higher than that of flesh. The reducing power and radical scavenging activities of peel were 11.98 ± 1.20 and 28.77 ± 0.62 μg/ml, while for flesh they were 24.38 ± 1.27 and 57.93 ± 1.84 μg/ml, respectively (less IC_50_ value shows the higher antioxidant activities). Ben Haj Koubaier et al. (2014) previously reported the DPPH radical scavenging activity (IC_50_) of root and stem of red beet as 5 and 47 μg/ml, respectively, which indicates that red beet root has a higher antioxidant activity than sugar beet. A comparison of Tables 1 and 3 also shows that the TPC and antioxidant capacity are well correlated for sugar beet extract. High positive correlation between phenolic compounds content and biological properties of some plant extracts has been reported in previous studies (Amiri et al., 2021; Ben Haj Koubaier et al., 2014; Sadeghnezhad et al., 2020).

**TABLE 3:** Antioxidant activity of the methanolic sugar extract of sugar beet and some phenolic standards (μg/ml)

Sample	Antioxidant activity		
	DPPH^·^ (IC_50_)	FRAP (IC_50_)	NOs (IC_50_)
Peel extract	28.77 ± 0.62	11.98 ± 1.20	88.17 ± 05.14
Flesh extract	57.93 ± 1.84	24.38 ± 1.27	119.17 ± 07.11
Gallic acid	32.68 ± 3.08	13.27 ± 0.41	126.63 ± 11.00
Epicatechin	17.22 ± 2.23	8.83 ± 0.25	96.08 ± 11.02
Rutin	25.36 ± 2.21	7.95 ± 0.74	116.99 ± 16.47
Trolox	53.61 ± 3.03	17.21 ± 0.69	155.56 ± 17.06
BHT	38.60 ± 1.10	11.66 ± 1.01	183.93 ± 12.95

Abbreviations: BHT, butylated hydroxytoluene; DPPH^·^, DPPH free radical scavenging activity; FRAP, ferric reducing antioxidant power; NOs, nitric oxide radical scavenging assay.

In the recent year, the study of bioactive compounds to prevent overproduction of NO has become a new research target, because excessive production of nitric oxide leads to various diseases such as inflammation, cancer, atherosclerosis, and diabetes mellitus (Taira et al., 2015). According to the results of Table 3, the sugar beet root parts (peel and flesh) exhibit high NOs activity, with IC_50_ values of 88.17 ± 5.14 and 119.17 ± 7.11 μg/ml, respectively. Comparing the NOs (IC_50_) of beet root extracts with other plant extracts shows that sugar beet had an interesting scavenging activity of nitric oxide radicals (Adiabouah Achy‐Brou & Billack, 2017; Lalegani et al., 2018; Oirere et al., 2016).

The beet extract potentials were also compared with the antioxidant activities of the related phenolics (gallic acid, epicatechin, and rutin) and synthetic antioxidants (Trolox and BHT) (Table 3). As can be seen, the peel extract showed a higher scavenging activity than synthetic antioxidants, indicating its promising biological benefits. The antioxidant capacity as measured by the DPPH֯ assay decreased in the order of epicatechin > rutin > peel extract > gallic acid > BHT > Trolox > flesh. In addition, the reducing power of the peel extract as measured by the FRAP assay (IC_50_: 11.98 ± 1.20 μg/ml) was close to the reducing power of BHT (IC_50_: 11.66 ± 1.01 μg/ml). The NOs activity of the standard synthetic antioxidants and related phenolics was also significantly lower than that of the peel extract. The high antioxidant activity of sugar beet may be due to the positive synergetic effect of bioactive compounds, existing in the peel extract (Ksouri et al., 2009).

### 3.4 Chelating ability of ions

**FIGURE 2:**
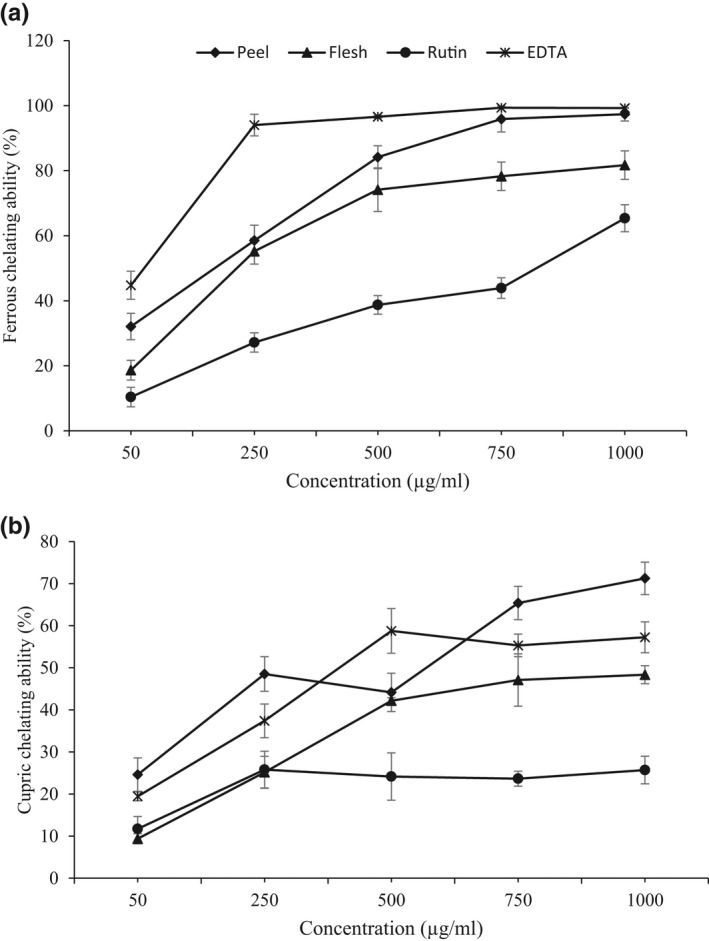
Chelating ability of different concentrations of sugar beet parts and standard solution (rutin and butylated hydroxytoluene (BHT)) on ferrous (a) and cupric ions (b).

Another proposed mechanism for the antioxidant activity of natural compounds is the chelation of transition metals (Fe, Cu, …), in which antioxidants suppress the formation of reactive species. Figure 2 (a) shows that the Fe^2+^‐chelating ability of sugar beet parts increases with concentration (50–100 μg/ml). At 250 μg/ml, chelating abilities of methanolic extracts of peel and flesh were 58.52 ± 4.70% and 55.21 ± 3.95%, and reached 97.36% and 81.7% at 1000 μg/ml, respectively. However, EDTA shows a high affinity binding of ferrous ions (Fe^2+^) and its value was 93.70 ± 3.33% at 250 μg/ml. Rutin shows a weak chelating ability for binding to ferrous ions and its value was 27.16 ± 2.94% at 250 μg/ml. Tohma et al. (2017) reported that water and ethanolic extract of ginger chelated 27.3% and 36.2% ferrous ions (Fe^2+^) at 10 μg/ml concentration. In another study, the metal‐chelating activity of the ethanolic extract from liquorice root was reported as 88% at 30 μg/ml (Tohma & Gulçin, 2010). Transition metals play an important role in the generation of reactive oxygen species in vivo via Fenton reactions (following equation), associating with many diseases such as cancers and heart problems (Santos et al., 2017).

$${\mathrm{Fe}}^{3+}+\cdot O_{2}^{-}\to {\mathrm{Fe}}^{2+}+O_{2}$$

$${\mathrm{Fe}}^{2+}+H_{2}O_{2}\to {\mathrm{Fe}}^{3+}+\cdot \mathrm{OH}+{\mathrm{OH}}^{-}$$

Therefore, the binding of these metals to the antioxidants (such as Fe^2+^‐polyphenol complexes) can decrease the subsequent oxidative damage and oxygen toxicity to cells. According to literature, the metal‐chelating ability of phenolic compounds is dependent on the presence of some functional groups such as hydroxyl and 4‐keto (Santos et al., 2017). They also claimed that the number and location of the functional groups play an important role in the exhibition of chelating activities. In the study conducted by Liu et al. (2015), it was found that peach peels (20–36 mg/ml) had higher metal‐chelating ability with lower IC_50_ value than its pulps (28–45 mg/ml). Generally, there are contradictory reports on the metal‐chelating ability of phenolic compounds and other substances under in vitro conditions. Some studies have showed that polysaccharides (e.g., fucoidans and alginates) and proteins were more effective than phenolics for the chelation of metal ions (Wang et al., 2010).

The Cu^2+^‐chelating results (Figure 2b) also showed that the tested samples had fewer ability to chelate cupric ions compared to the ferrous ions. At 250 μg/ml, Cu^2+^‐chelating abilities of peel and flesh methanolic extracts were 48.52 ± 4.10% and 25.16 ± 3.80%, and reached 71.25 ± 3.84% and 48.35 ± 2.12% at 1000 μg/ml, respectively. Říha et al. (2014) stated that the most efficient Cu^2+^‐chelation sites in flavonoids were the 5,6,7‐trihydroxyl group in flavones and the 3‐hydroxy‐4‐keto group in flavonols. EDTA shows a weak affinity binding of cupric ions (Cu^2+^) and its value was 37.40 ± 4.00% at 250 μg/ml. Rutin also showed a relatively weak Cu^2+^‐chelating activity (25.16 ± 4.36% at 250 μg/ml) and did not change significantly with increasing its concentration (250–1000 μg/ml). Mendoza‐Wilson et al. (2016) reported that the Cu^2+^‐chelating activities of the aqueous and ethanolic extracts from apple skin were almost identical and equal to 66.83% and 67.40%, respectively, at a concentration of 0.5 mg/ml. Therefore, based on our results and mentioned studies, it can be stated that sugar beet has a high potential for chelating transition metals (Fe^2+^ and Cu^2+^) and providing health beneficial effects.

## 4 CONCLUSIONS

In the current work, we evaluated the phenolic composition and the biological properties of sugar beet as a medicinal root. Ten phenolic compounds have been separated and determined in various parts of sugar beet by RP‐HPLC‐UV (reversed‐phase high‐performance liquid chromatography with ultraviolet detection). Gallic acid, epicatechin, and quercetin‐3‐O‐rutinoside were the dominant phenolics that were detected in sugar beet. The root peel extracts had a higher amount of bioactive compounds and biological properties (antioxidant and chelating activity) compared to the flesh extract. In addition, a high correlation was observed between TPC and antioxidant capacity of the sugar beet extract. As a result, it can be said that sugar beet is potentially a rich source of bioactive compounds with promising biological effects that need to be investigated further in the future.

## CONFLICT OF INTEREST

The authors have declared no conflict of interest.

## Data Availability

Data will be made available on request from the authors.
